# Tryptophan Metabolites Are Associated With Symptoms and Nigral Pathology in Parkinson's Disease

**DOI:** 10.1002/mds.28202

**Published:** 2020-07-25

**Authors:** Patrick L. Heilman, Ernest W. Wang, Mechelle M. Lewis, Stanislaw Krzyzanowski, Colt D. Capan, Amanda R. Burmeister, Guangwei Du, Martha L. Escobar Galvis, Patrik Brundin, Xuemei Huang, Lena Brundin

**Affiliations:** ^1^ Center for Neurodegenerative Science Van Andel Institute Grand Rapids Michigan USA; ^2^ Department of Neurology Penn State University‐Milton S. Hershey Medical Center Hersey Pennsylvania USA; ^3^ Department of Pharmacology Penn State University‐Milton S. Hershey Medical Center Hersey Pennsylvania USA; ^4^ Department of Neurosurgery Penn State University‐Milton S. Hershey Medical Center Hersey Pennsylvania USA; ^5^ Department of Radiology Penn State University‐Milton S. Hershey Medical Center Hersey Pennsylvania USA; ^6^ Department of Kinesiology Penn State University‐Milton S. Hershey Medical Center Hersey Pennsylvania USA; ^7^ Division of Psychiatry & Behavioral Medicine Michigan State University College of Human Medicine Grand Rapids Michigan USA

**Keywords:** cerebrospinal fluid, inflammation, kynurenine metabolites, Parkinson's disease, tryptophan

## Abstract

**Background:**

The objective of this study was to determine whether neurotoxic kynurenine metabolites, induced by inflammation, in plasma and cerebrospinal fluid (CSF) are associated with symptom severity and nigral pathology in Parkinson's disease (PD).

**Methods:**

Clinical and MRI data were obtained from 97 PD and 89 controls. We used ultra‐performance liquid chromatography to quantify kynurenine metabolites and high‐sensitivity multiplex assays to quantify inflammation in plasma and CSF. We evaluated group‐wise differences as well as associations between the biomarkers, motor and nonmotor symptoms, and nigral R2* (MRI metric reflecting iron content).

**Results:**

PD subjects had >100% higher 3‐hydroxykynurenine and 14% lower 3‐hydroxyanthranilic acid in plasma. The 3‐HK in plasma was closely associated with both symptom severity and disease duration. PD subjects also had 23% lower kynurenic acid in the CSF. Higher CSF levels of the excitotoxin quinolinic acid were associated with more severe symptoms, whereas lower levels of the neuroprotective kynurenic acid were linked to olfactory deficits. An elevated quinolinic acid/picolinic acid ratio in the CSF correlated with higher R2* values in the substantia nigra in the entire cohort. Plasma C‐reactive protein and serum amyloid alpha were associated with signs of increased kynurenine pathway activity in the CSF of PD patients, but not in controls.

**Conclusions:**

In PD, the kynurenine pathway metabolite levels are altered in both the periphery and the central nervous system, and these changes are associated with symptom severity. Replication studies are warranted in other cohorts, and these can also explore if kynurenine metabolites might be PD biomarkers and/or are involved in PD pathogenesis. © 2020 The Authors. *Movement Disorders* published by Wiley Periodicals LLC on behalf of International Parkinson and Movement Disorder Society.

Parkinson's disease (PD) is a common, progressive movement disorder that also features debilitating nonmotor symptoms. The neurodegenerative features of PD include the loss of dopaminergic neurons in the substantia nigra pars compacta, the formation of α‐synuclein‐containing Lewy bodies and increased neuroinflammation.^1^, ^2^ Current therapies only temporarily reduce PD symptoms and do not stop disease progression. The mechanisms responsible for neuronal death in PD are not fully understood, which has made the development of disease‐modifying therapies a challenge. Furthermore, there is a lack of biomarkers that could predict PD development or reflect disease severity.

Increased metabolism of tryptophan via the kynurenine pathway (Fig. 1) is a consequence of inflammation.^3^, ^4^ A number of intermediate metabolites of this pathway have potent neuroactive and inflammatory properties. Quinolinic acid (QUIN) is an N‐methyl‐D‐aspartate (NMDA)–receptor agonist and excitotoxin. In the presence of high levels of QUIN, overactivation of glutamate receptors leads to increased cytoplasmic levels of Ca^2+^, which may initiate a range of toxic processes. In addition to its direct action at the NMDA receptor, QUIN can cause increased neuronal glutamate release, decreased glutamate uptake by astrocytes, and activation of microglia and neuroinflammation.^5^ Another kynurenine pathway metabolite is 3‐hydroxykynurenine (3‐HK), which can induce cell death by the generation of free radicals and oxidative stress and can act in synergy with QUIN.^6^ Other metabolites, such as kynurenic and picolinic acids, counteract the effects of QUIN and 3‐HK and thereby can be neuroprotective.^4^ Because changes in the kynurenine pathway metabolites can lead to neurodegeneration, this metabolic pathway has been suggested to be part of the PD pathogenesis.^7^ Multiple lines of evidence based on both clinical observations and findings in experimental models support this idea. Several large‐scale genome‐wide association studies^8^, ^9^ have identified single‐nucleotide polymorphisms associated with PD risk in regulatory regions of 2‐amino‐3‐carboxymuconic‐6‐semialdehyde decarboxylase (ACMSD), an enzyme in the kynurenine pathway that directly regulates the production of QUIN. Moreover, point mutations^10^ and deletions^11^ within the *ACMSD* gene itself have been found in sporadic PD and a familial syndrome with tremor and parkinsonism, respectively. Recent studies have detected activation of the kynurenine pathway in the plasma of PD patients,^12^, ^13^, ^14^ but did not reveal if the changes were associated with symptom severity, ongoing systemic inflammation, or changes in brain as detected by MRI imaging in PD.

Thus, in the present study we examined a large and clinically well‐characterized cohort to establish how/if the levels of kynurenine metabolites and inflammatory factors in plasma and CSF differ between PD subjects and age‐ and sex‐matched healthy controls. We hypothesized that PD subjects would exhibit increased activation of the kynurenine pathway with an accumulation of the neurotoxic metabolites QUIN and 3‐HK and that these changes would associate with the severity of clinical symptoms as well as with neuroimaging metrics reflecting PD‐related neuropathology in the substantia nigra.

## Methods

### Participants

We obtained clinical and MRI data from 100 PD and 90 control subjects who participated in the National Institute of Neurological Disorders and Stroke PD Biomarker Program (NINDS PDBP) at Pennsylvania State University between 2012 and 2015. PD diagnosis was confirmed by movement disorders specialists.^15^, ^16^ Three patients were excluded due to other neuropathological diagnoses postmortem, leaving 97 patients. One control received a postmortem neurological diagnosis and was excluded, leaving 89 controls. All 186 included participants were free of major medical issues and neurological conditions other than PD. Demographic data are provided in Supplemental Table 1. Disease duration was defined as years between date of first PD diagnosis by a medical professional and the study visit date.

### Clinical Assessments

The NINDS PDBP used NINDS common data elements for collection of clinical data. We used the UPDRS scores (obtained for PD participants in the “on‐medication” state) to assess nonmotor and motor severity. Montreal Cognitive Assessment (MoCA)^17^ and University of Pennsylvania Smell Identification Test (UPSIT)^18^ scores were used to assess cognitive functioning and olfaction.

### Blood and CSF Sampling

Biological samples were drawn in the morning before 10 am. Fasting blood was obtained in BD Vacutainer Glass Blood Collection Tubes with K_3_‐ethylenediaminetetraacetic acid (Fisher Scientific, Hampton, NH), centrifuged at 1700*g* for 10 minutes, followed by aspiration, ensuring that none of the buffy coat contaminated the plasma. CSF samples were available for a subset (30 controls and 25 PD patients). Lumbar punctures were performed by an experienced neurologist following sterile PDBP procedures that used a 24/22G Sprotte atramatic, introducer, and 22G hypodermic needles, along with a plastic syringe (6 mL) to draw the CSF. Within 15 minutes of collection, the samples were transferred to 15 mL of conical tubes and centrifuged at 2000*g* for 10 minutes at room temperature. For both plasma and CSF, 1‐mL aliquots were banked at Pennsylvania State University in a −80°C freezer with CO_2_ backup and 24/7/365 monitoring. For the current project, the samples were stored at −80°C for 4–7 years, then thawed once, when aliquoted for shipment to the Van Andel Institute on dry ice for analysis.

### Preparation of Plasma and CSF Samples for UPLC Analysis

Plasma samples were mixed with internal standard diluent and centrifuged. The supernatant was removed and dried under reduced pressure conditions by Savant Speedvac SPD131DDA (Thermo Fisher Scientific, Waltham, MA). Samples were resuspended in 0.6% formic acid in Milli‐Q water, centrifuged through a COSTAR Spin‐X 0.22‐μm filter tube and transferred to an amber vial (Agilent) containing a glass insert. CSF samples were combined 1:2 with a solution containing 500 nM ^2^H_4_‐KYN, ^2^H_5_‐KA, ^2^H_4_‐picolinic acid (PIC), ^13^C_6_‐nicotinamide (NTA), ^2^H_3_‐QUIN, ^13^C_2_.^15^N‐3HK, ^2^H_3_‐3HAA (5000 nM ^2^H_3_‐TRP) 1.2% formic acid in Milli‐Q water, vortexed and centrifuged through a COSTAR Spin‐X 0.22‐μm filter tube, and transferred to an amber vial (Agilent) containing a glass insert.

### Quantification of Kynurenine Metabolites

**FIG. 1:**
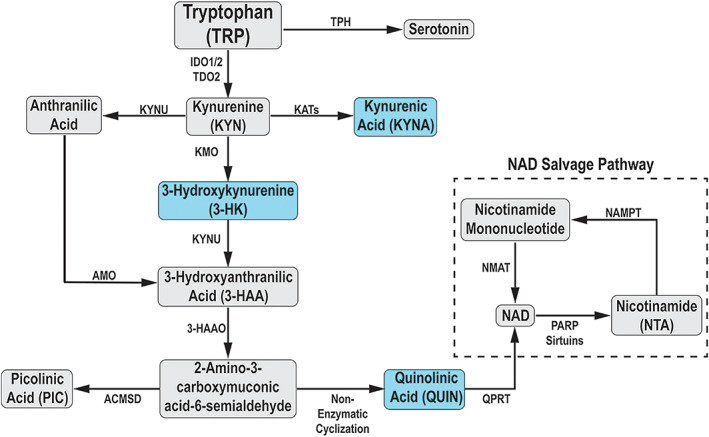
Simplified schematic of the kynurenine pathway. IDO, indoleamine 2,3‐dioxygenase; TDO, tryptophan 2,3‐dioxygenase; KAT, kynurenine amino transferase; KYNU, kynureninase; KMO, kynurenine 3‐monooxygenase; 3‐HAO, 3‐hydroxyanthranilic acid 3,4‐dioxygenase; ACMSD, α‐amino‐β‐carboxymuconate‐ε‐semialdehyde decarboxylase; QPRT, quinolinate phosphoribosyl transferase; NAD, nicotinamide dinucleotide. Metabolites shaded in blue are known to have neuroactive properties. Metabolites with abbreviations were analyzed in this study. [Color figure can be viewed at wileyonlinelibrary.com]

Kynurenine pathway metabolites (KYN, KYNA, 3‐HK, 3‐HAA, QUIN, PIC, NTA), tryptophan, and serotonin (see Fig. 1) were quantified from plasma and CSF using a Waters Acquity ultra‐high‐performance liquid chromatography (UPLC) I class and Xevo triple‐quadrupole mass spectrometry system. Ten‐microliter samples were injected into an Acquity HSS T3 column conjugated with a Vanguard HSS T3 guard column and eluted using a mobile phase, 0.6% formic acid in Milli‐Q water (Sslvent A), and 0.6% formic acid in analytical‐grade methanol (solvent B), at an isocratic flow rate of 0.3 mL/min for plasma samples and 0.4 mL/min for CSF samples. Intra‐assay coefficients of variability (CV) for plasma analytes were: TRP, 4.7%; KYN, 3.7%; KYNA, 4.7%; 3‐HK, 2.5%; 3‐HAA, 6.1%; QUIN, 2.6%; PIC, 4.1%; NTA, 6.0%; and 5‐HT, 5.5%. Intra‐assay CVs for CSF analytes were: TRP, 0.8%; KYN, 2.4%; KYNA, 14.6%; 3‐HK, 9.1%; QUIN, 5.0%; PIC, 5.2%; and NTA, 12.9%. Data for CSF PIC was discarded for one control due to technical error. Lower limits of detection (LLOD) in both plasma and CSF were 0.05 nM for all metabolites except TRP, which was 5 nM.

### Quantification of Inflammatory Factors

C‐reactive protein (CRP) and serum amyloid alpha (SAA) levels were analyzed on a Meso Scale Discovery Sector 6000 imager, according to the manufacturer's instructions (Meso Scale Diagnostics LLC, Rockville, MD). All samples were run in duplicate, and the average value of each sample was used for statistical analysis. Intra‐assay CVs for the analytes measured in plasma were: CRP, 5.2%; and SAA, 4.2%. Intra‐assay CVs for the analytes measured in CSF were: CRP, 4.2%; and SAA, 3.9%. Lower limit of quantification (LLOQ) for analytes measured in plasma were: CRP, 1.68 pg/mL; and SAA, 8.98 pg/mL. LLOQ for analytes measured in CSF were: CRP, 2.25 pg/mL; and SAA, 13.80 pg/mL.

### MRI Acquisition and Analysis

As part of the NINDS PDBP at Penn State, T1‐weighted, T2‐weighted, and multigradient‐echo magnetic resonance images were acquired on a 3T Siemens scanner for each subject. Detailed scan parameters are described in a previous study.^19^ Mean R2* values from the substantia nigra pars compacta (including both sides) were calculated for individual subjects. For the purposes of this study, R2* values that were generated previously were used for correlation analysis with the kynurenine pathway metabolites.

### Statistical Analyses

**FIG. 2:**
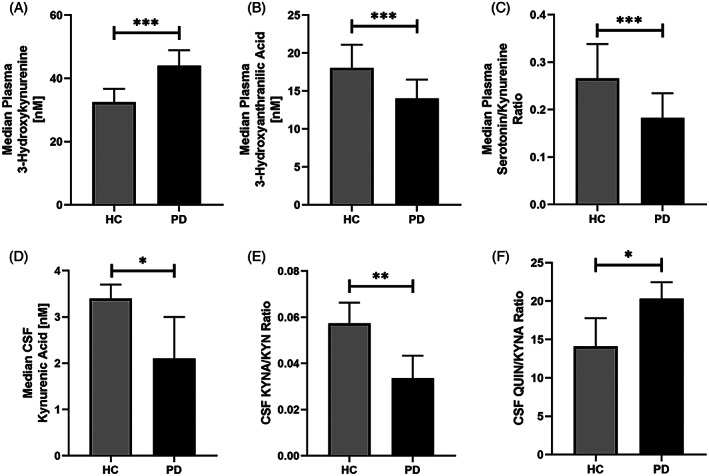
Comparison of kynurenine metabolite levels in HC and PD subjects. Median plasma levels of 3‐hydroxykynurenine (**A**), 3‐hydroxyanthranilic acid (**B**), serotonin/kynurenine ratio (**C**), and CSF kynurenic acid levels (**D**), kynurenic acid/kynurenine ratio (**E**), and quinolinic acid/kynurenic acid ratio (**F**) in healthy controls (n = 89) and Parkinson's disease subjects (n = 97). Error bars represent 95% confidence interval of the median. Significance was determined using the Mann‐Whitney *U* test. ***P < 0.001, **P < 0.005, *P < 0.05.

**FIG. 3:**
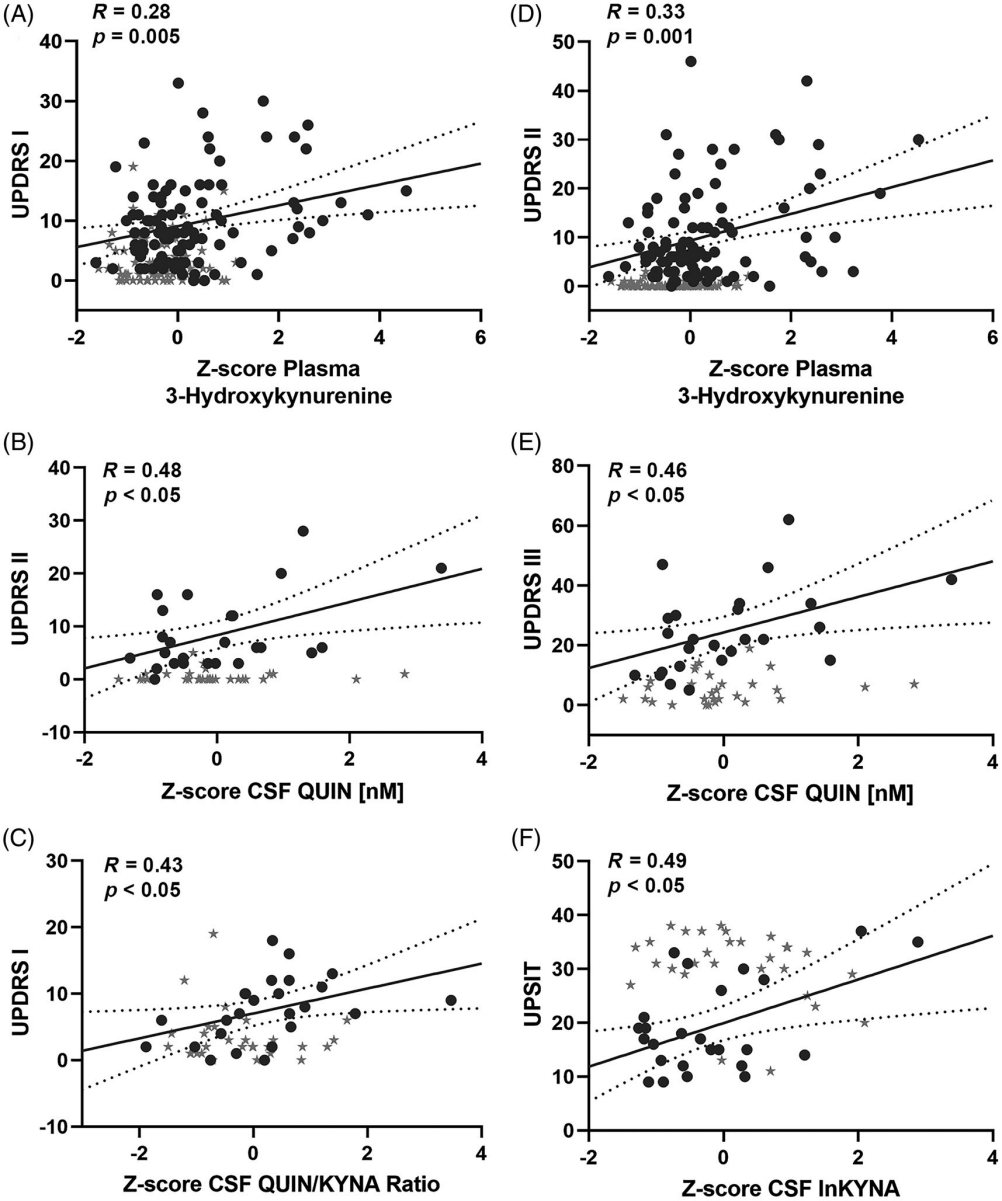
Kynurenine metabolite correlations with UPDRS scores. Scatterplot correlations between (**A**) plasma 3‐hydroxykynurenine (3‐HK) levels and Unified Parkinson's Disease Rating Scale (UPDRS) I scores, (**B**) plasma 3‐HK and UPDRS II scores, (**C**) CSF quinolinic acid (QUIN) levels and UPDRS II scores, (**D**) CSF QUIN levels and UPDRS III scores, (**E**) CSF QUIN/KYNA ratio and UPDRS scores, and (**F**) CSF kynurenic acid (KYNA) levels and UPSIT scores in PD subjects (n = 25). The plotted Pearson correlation coefficients and *P* values derive from the PD cohort after adjusting for age and sex. Filled circles represent patients, and stars denote healthy control data points. Dashed lines indicate the 95% confidence interval for the line of best fit.

**FIG. 4:**
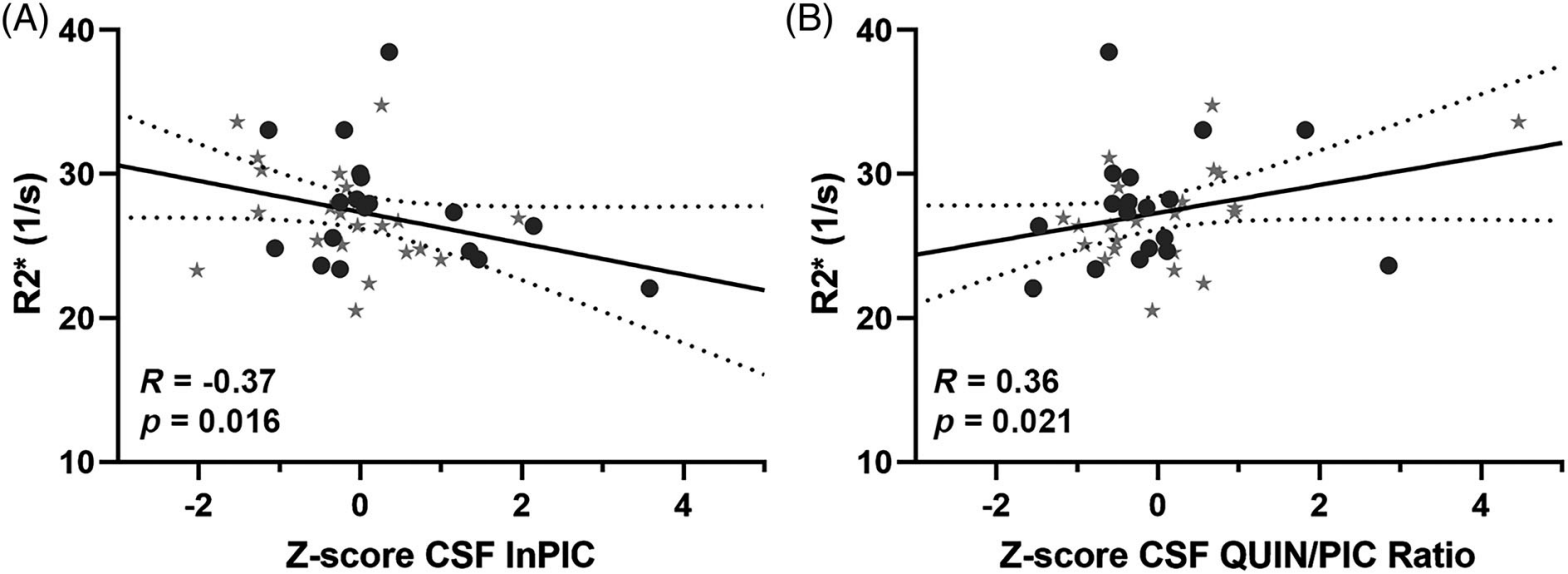
Correlation of R2* values with CSF kynurenine metabolites. Scatterplot correlations between (**A**) CSF picolinic acid (PIC) levels and (**B**) CSF QUIN/PIC ratios and R2* values in the substantia nigra pars compacta. Data from controls are plotted as stars and from patients as filled circles. Pearson correlation coefficients and *P* values are from partial correlation analysis performed after adjusting for age and sex on the entire data set (n = 41). Dashed lines indicate the 95% confidence interval for the line of best fit.

**FIG. 5:**
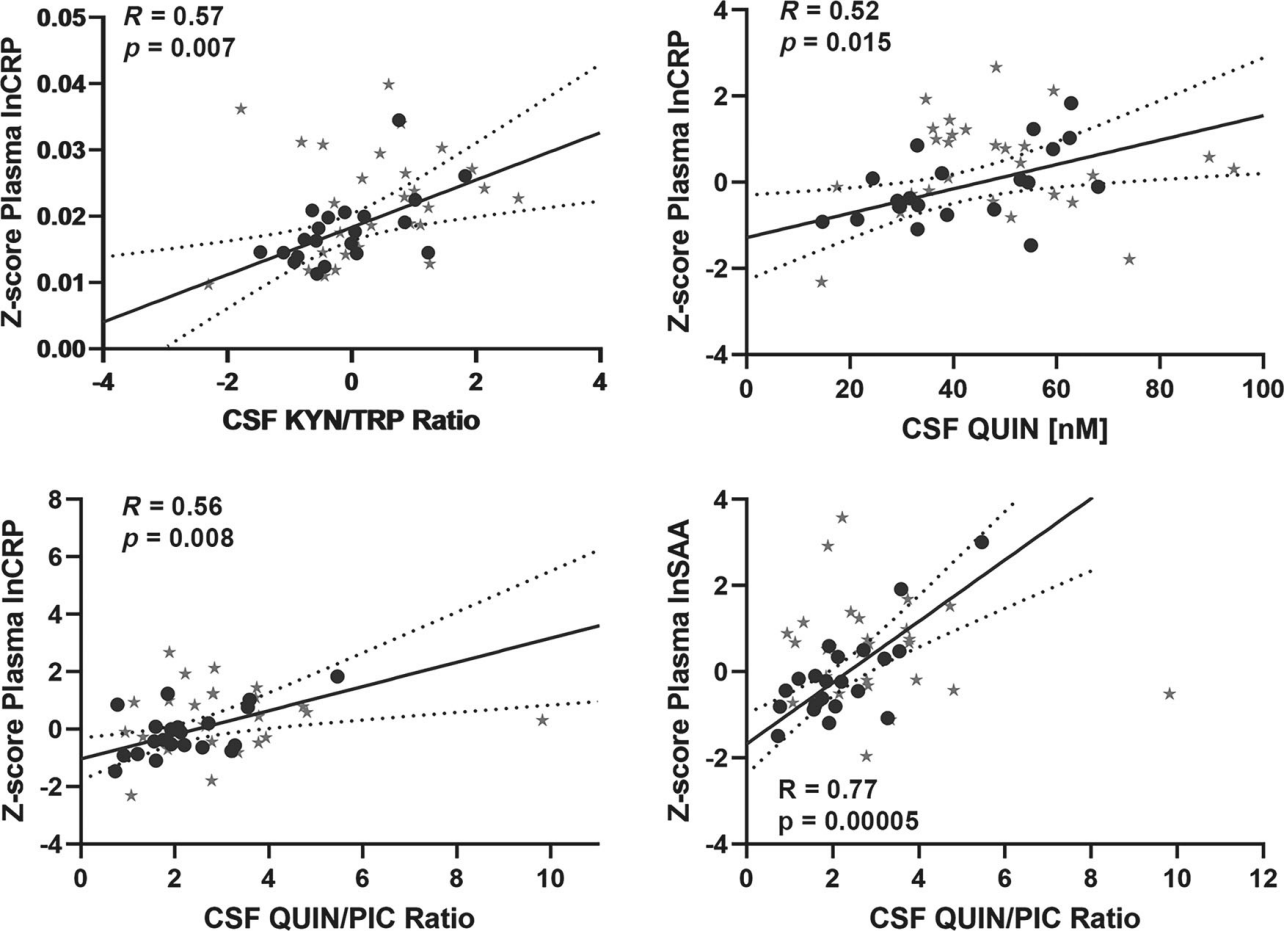
Correlation of peripheral inflammatory markers and CSF kynurenine metabolites in PD patients. Scatterplot correlations between (**A**) plasma C‐reactive protein (CRP) levels and CSF quinolinic acid (QUIN) levels, (**B**) plasma CRP levels and CSF kynurenine/tryptophan (KYN/TRP) ratio, (**C**) plasma CRP levels and CSF QUIN/PIC ratio, (**D**) plasma serum amyloid alpha (SAA) levels and CSF QUIN/PIC ratio in PD subjects (n = 25). Data from healthy controls are represented by stars and patients by filled circles. The plotted Pearson correlation coefficients and *P* values after adjusting for age and sex, are for the patient cohort analyzed separately. Dashed lines indicate the 95% confidence interval for the line of best fit.

Statistical analyses were performed using IBM's Statistical Package for the Social Sciences (SPSS v.25). Comparisons of demographic variables were made using Student *t* tests for continuous variables and chi‐square tests for categorical variables. Group‐wise comparisons between PD subjects and controls were performed using the Mann‐Whitney *U* test on the raw data, visualized in Figure 2. Biomarkers were transformed by the natural logarithm to enable adjustment for sex and age, and the association with clinical and imaging metrics was analyzed using Pearson's correlation. The age‐ and sex‐adjusted *Z* scores were generated from linear regression models. As exploratory outcomes, we determined the relationship between plasma and CSF kynurenine metabolites and inflammatory markers, as well as between plasma and CSF biomarkers and disease duration, by Pearson's correlation using the age‐ and sex‐adjusted *Z* scores. To correct for multiple comparisons, significance was determined using a false discovery rate (FDR) at 10% as described by Benjamini‐Hochberg.^20^ This rate was selected a priori to improve our probability of detecting true differences in the exploratory analyses, understanding that ~10% of them will still be false‐positives. Future research will help to clarify which, if any, of these identified markers was a false discovery, but for now yields a comprehensive set of screened markers to focus on in subsequent studies. Both original and FDR‐adjusted *P* values are presented in Supplemental Tables 3 and 4. For visualization, *Z* scores are plotted in Figures 3, 4, 5.

### Standard Protocol Approvals, Registrations, and Patient Consents

All participants gave written informed consent in accordance with the Declaration of Helsinki. The study was approved by the Pennsylvania State University Hershey Institutional Review Board and the Van Andel Institute Review Board.

### Data Availability

All data generated or analyzed during this study are included in this published article.

## Results

### Demographics

The demographics of the plasma cohort are described in Supplemental Table 1. Subjects and controls did not differ in respect to age (66.9 vs 66.6 years), body mass index (27.5 vs 28.1 kg/m^2^), years of education (15 vs 15 years), or sex (49% women vs 53% women). As expected, PD subjects had significantly higher UPDRS I–III scores, lower MoCA and UPSIT scores, and higher R2* values compared with controls (Supplemental Table 1).

### Plasma Kynurenine Pathway Metabolites Are Changed in PD

PD subjects had significantly higher plasma levels of 3‐HK compared with controls (n = 185, *P* = 0.000005; Fig. 2A) and lower levels of the subsequent metabolite in the pathway, 3‐HAA (*P* = 0.008; Fig. 2B), as well as lower NTA (*P* = 0.05), an end product of the kynurenine pathway. There were no significant differences in the absolute plasma levels of TRP, KYN, KYNA, QUIN, or PIC between PD and control subjects (data shown in Supplemental Table 2). PD subjects also displayed a lower 5‐HT/KYN ratio (*P* = 0.001; Fig. 2C).

### CSF Metabolites Are Altered in PD Versus Controls

PD subjects had significantly lower levels of the neuroprotective metabolite KYNA in the CSF compared with controls (n = 55, *P* = 0.029; Fig. 2D). Furthermore, the KYNA/KYN ratio was lower in PD (*P* = 0.002; Fig. 2E), indicating a reduced formation of KYNA from KYN and instead a shift toward an increased neurotoxic ratio, QUIN/KYNA (*P* = 0.046; Fig. 2F). There were no differences in the absolute CSF levels of TRP, KYN, 3‐HK, QUIN, PIC, or NTA between PD and controls (Supplemental Table 2).

### Association Between Plasma Analytes and Symptom Severity

We assessed the capacity of peripheral levels of 3‐HK and 3‐HAA to reflect symptom severity by establishing the association between the age‐ and sex‐adjusted variables with UPDRS I–III symptom severity. Plasma 3‐HK was significantly associated with UPDRS I (*R* = 0.28, *P* = 0.005; Fig. 3A), UPDRS II (*R* = 0.33, *P* = 0.001; Fig. 3B), and UPDRS III (*R* = 0.24, *P* = 0.02) in patients (n = 97). The 3‐HK was not associated with symptoms within the controls. The 3‐HAA was not linked to any symptoms in patients or controls.

### Association Between CSF Analytes and Symptom Severity

We next assessed the association between age‐ and sex‐adjusted CSF levels of kynurenines and symptoms. In PD patients (n = 25), higher CSF levels of QUIN were significantly associated with higher total UPDRS II (motor aspects of daily living [ADLs]) and UPDRS III (motor examination) scores (*R* = 0.48, *P* = 0.015; Fig. 3C; and *R* = 0.46, *P* = 0.021; Fig. 3D, respectively). Increased activation of the first step of the pathway, the KYN/TRP ratio, was also associated with higher ADL and motor scores (UPDRS II: *R* = 0.41, *P* = 0.042; UPDRS III: *R* = 0.40, *P* = 0.05). Higher scores on these scales indicate more severe symptoms.

The QUIN/PIC (*R* = 0.66, *P* = 0.0003) and QUIN/3‐HK (*R* = 0.52, *P* = 0.008) ratios were both strongly associated with increasing severity of ADL symptoms in the PD subjects (UPDRS II total score). The QUIN/KYNA ratio (*R* = 0.43, *P* = 0.034; Fig. 3E), often denoted the neurotoxic ratio,^21^ was linked to higher scores on the nonmotor aspects of activities of daily living symptoms (UPDRS I total score). None of these correlations were present in the controls (data for patients and controls shown in Fig. 3).

Higher CSF levels of KYNA were associated with higher UPSIT scores. Higher scores on this scale indicate better olfaction, which thus correlated with high levels of the neuroprotective KYNA (*R* = 0.49, *P* = 0.013; Fig. 3F). The correlation was not detected in controls.

### Kynurenine Metabolites and R2* in the Substantia Nigra

We have previously shown that elevated R2* in the substantia nigra pars compacta, reflecting iron content, is associated with PD progression^19^ and related pathological processes^22^ in our cohort. In the entire cohort (n = 41), there was a correlation between lower levels of the iron chelator PIC and higher R2* in the substantia nigra pars compacta (*R* = −0.37, *P* = 0.016; Fig. 4A). Similarly, an increasing QUIN/PIC ratio was associated with higher R2* (*R* = 0.36, *P* = 0.021; Fig. 4B). When separated into controls and patients, the data were significant only in the 23 controls (PIC *R* = −0.47, *P* = 0.023; and QUIN/PIC *R* = 0.55, *P* = 0.006).

### Changes of Inflammatory Markers in PD Plasma and CSF

Median levels of the acute‐phase proteins were lower in both CSF and plasma of the PD patients compared with controls (Supplemental Table 2). CRP was significantly lower in the plasma (*P* = 0.001), and there was a trend toward lower SAA in both the CSF (*P* = 0.07) and plasma (*P* = 0.08) of PD patients (Supplemental Table 2).

Because inflammation is known to activate the kynurenine pathway, we examined the association between inflammatory markers in plasma with the kynurenine metabolites in the CSF of PD subjects (n = 25) and controls (n = 30); see Supplemental Table 3. In the PD patients, we found that plasma CRP and SAA were associated with the CSF KYN/TRP ratio (*R* = 0.57, *P* = 0.007; Fig. 5A; and *R* = 0.52, *P* = 0.016; respectively), suggesting a potential connection between peripheral inflammation and central tryptophan metabolism in PD subjects. Higher plasma CRP and SAA were also linked with higher CSF QUIN (*R* = 0.52, *P* = 0.015; Fig. 5B; and *R* = 0.50, *P* = 0.02, respectively) and with the QUIN/PIC ratio (*R* = 0.56, *P* = 0.008; Fig. 5C; and *R* = 0.77, *P* = 0.00005; Fig. 5D, respectively). In contrast, there were no associations between plasma levels of inflammatory markers and kynurenine metabolites in the CSF of controls (Supplemental Table 3). Plasma and CSF CRP and SAA were closely associated across the blood–brain barrier in both patients and controls (patients: CRP in plasma vs CSF, *R* = 0.90, *P* = 0.000005; SAA in plasma vs CSF, *R* = 0.82, *P* = 0.000002).

### Association of Biomarkers With Disease Duration

We investigated the association between PD disease duration and the biomarkers, adjusted for sex and age. Plasma 3‐HK (*R* = 0.33, *P* = 0.001) as well as the CSF levels of the acute‐phase proteins SAA (*R* = 0.71, *P* = 0.000075) and CRP (*R* = 0.51, *P* = 0.01) correlated with disease duration. Adjusting for sex only did not alter these results (not shown). The correlation with disease duration is presented in full in Supplemental Table 4.

## Discussion

We found that the metabolite patterns in both CSF and plasma from PD patients were consistent with inflammatory activation of the kynurenine pathway. Specifically, PD subjects had higher amounts of the neurotoxic metabolite 3‐HK in plasma and an increased QUIN/KYNA ratio in the CSF compared with controls. The acute‐phase proteins CRP and SAA were associated with the induction of the pathway (KYN/TRP ratio) and QUIN levels in the CSF. Higher levels of neurotoxic kynurenine metabolites in both plasma and CSF correlated with more severe symptoms. Together, these data suggest that kynurenine metabolites may serve as novel biomarkers and/or are involved in the PD pathological process.

We observed higher plasma 3‐HK in PD subjects, in line with a recent study that found elevated 3‐HK in a small group of PD subjects with dyskinesias (n = 12).^12^ The majority of patients in our study were free from dyskinesias, suggesting that elevated plasma 3‐HK is not a feature exclusively of PD subjects with dyskinesias. We also found that plasma 3‐HK was associated with disease duration and symptom severity on UPDRS I–III, suggesting that 3‐HK might serve as a peripheral biomarker for PD severity and/or progression. This finding is also interesting in the context of PD pathogenetic mechanisms because 3‐HK has neurotoxic properties and induces mitochondrial dysfunction and oxidative stress.^23^, ^24^, ^25^, ^26^ We also found lower 3‐HAA, a downstream product of 3‐HK in the kynurenine pathway. This suggests that the accumulation of 3‐HK could result from reduced activity of kynureninase, the enzyme that converts 3‐HK to 3‐HAA. In spite of the changes in peripheral 3‐HK, we did not find any alterations in 3‐HK in CSF. This could indicate that the peripheral and central compartments differ in 3‐HK production and metabolism, or be because the number of CSF samples was smaller in our study.

In PD CSF samples, we detected a reduction of the neuroprotective metabolite KYNA.^27^, ^28^ This result is consistent with earlier smaller studies showing reduced plasma and CSF KYNA^14^, ^29^ and brain‐region‐specific reductions in KYNA in postmortem PD brain tissue.^30^ Because we also observed an increased QUIN/KYNA ratio in the CSF of PD subjects, the reduced KYNA could result from an increased metabolic flux through the QUIN‐producing branch of the kynurenine pathway. This shift in enzymatic activity at the expense of KYNA and serotonin (5‐HT) is known to occur during inflammatory and infectious conditions.^31^, ^32^ Although 5‐HT is produced from tryptophan, it is not a kynurenine pathway metabolite (Fig. 1). Consistent with the idea of shifting enzymatic activities between the serotonin‐kynurenine pathway, our patients also had a lower 5‐HT/KYN ratio in plasma compared with controls.

We did not observe elevated levels of acute‐phase proteins in PD, but CSF CRP and SAA levels correlated with disease duration, even when correcting for age, suggesting that inflammation is associated with disease progression. In addition, the acute‐phase reactants correlated closely between plasma and CSF in both PD patients and controls. This is interesting because the communication of the acute‐phase reactants across the blood–brain barrier is still poorly understood. Intriguingly, only in PD patients did the acute‐phase proteins correlate with the CSF QUIN, QUIN/PIC, and KYN/TRP ratios, suggesting a disease‐specific kynurenine pathway activation. The mechanism underlying this is unclear, and we speculate that the kynurenine pathway is more sensitive to activation by inflammation in PD.

Our study is the first to describe an association between CSF kynurenine metabolites and PD motor and nonmotor symptoms. We and others previously reported that subjects with depression and suicidality exhibit inflammation and increased QUIN/KYNA ratios.^31^, ^32^, ^33^, ^34^ Up to 90% of PD subjects exhibit depression or anxiety, and further studies are needed to investigate whether inflammation and kynurenine metabolites are directly involved in the specific psychiatric symptoms of PD.^35^, ^36^ Furthermore, our study is the first to explore the potential relationship between kynurenine metabolites in CSF and the neuroimaging metric R2*. In PD, R2* was elevated in the substantia nigra, reflecting elevated iron content.^19^, ^22^ When examining both PD and control subjects together, we found that the R2* values in the substantia nigra pars compacta correlated with higher CSF QUIN/PIC ratios. The QUIN/PIC ratio is regulated by the enzyme ACMSD generating PIC (at the expense of QUIN), which is a well‐known iron chelator, and therefore reduced ACMSD activity could contribute to the iron accumulation in the substantia nigra.^37^

The strengths of our study include a clinically well‐characterized cohort, measures of kynurenine metabolites and inflammatory markers in both plasma and CSF, and imaging data from the substantia nigra. As a limitation, this cross‐sectional study was associative, and therefore causality could not be demonstrated. In addition, the PD subjects were on medication and were evaluated in the ON state, as defined per the PDBP study protocol. Therefore, we could not rule out that the observed effects were impacted by medication. Finally, because of the number of analyses performed, there is always a risk of reporting spurious results. Thus, replication in an independent cohort is necessary, as well as studies of kynurenine metabolites in medication‐free subjects with early PD or in the defined OFF state.

In summary, we found evidence of an inflammation‐associated activation of the kynurenine pathway in both plasma and CSF in PD patients. We observed altered absolute levels of 3‐HK, 3‐HAA, and KYNA in PD, together with changes in several neurotoxic/neuroprotective ratios, indicating that tryptophan metabolism is disrupted in PD. In addition, we found that plasma and CSF levels of kynurenine metabolites were closely associated with worsening of symptoms in PD. Further replication studies are warranted, and these can also explore if kynurenine metabolites can serve as novel biomarkers and/or are involved in PD pathogenesis, which might lead to the identification of novel therapeutic targets.

## Supporting information


**SUPPLEMENTAL TABLE 1** Subject demographics.
MoCA, Montreal Cognitive Assessment; UPSIT, University of Pennsylvania Smell Identification Test; R2*, relaxation rates in the substantia nigra pars compacta; N/A; not available.
All values are reported as mean ± SD.^1^
^a^Student *t* test.
^b^Chi‐square test.
^c^ANCOVA, with age and sex as covariates.

**SUPPLEMENTAL TABLE 2** Median levels of metabolites and inflammatory markers. All values are reported as Median (IQR).
^a^Mann‐Whitney *U* test.

**SUPPLEMENTAL TABLE 3** Correlations over the blood–brain barrier (BBB)
Pearson correlation coefficients and *P* values after adjusting for sex and age.
FDR‐adjusted *P* values are listed in separate columns.

**SUPPLEMENTAL TABLE 4** Correlation of biological analytes with disease duration in years. Pearson correlation coefficients and *P* values after adjusting for sex and age. FDR‐adjusted *P* values are listed in separate columns.

## Appendix 1 1

**Table:**

Name	Location	Role	Contribution
Patrick L. Heilman, PhD	Van Andel Institute, Center For Neurodegenerative Sciences, Grand Rapids, MI	Author	Data collection, data analysis, drafting of the initial article
Ernest W. Wang	Penn State University, Milton S. Hershey Medical Center, Hershey, PA	Author	Data analysis, review of statistical analysis, critical review of the article
Mechelle M. Lewis, PhD	Penn State University, Milton S. Hershey Medical Center, Hershey, PA	Author	Study concept and design, clinical data collection, clinical data analysis, review of statistical analysis, critical review of the article
Stanislaw Krzyzanowski	Van Andel Institute, Center For Neurodegenerative Sciences, Grand Rapids, MI	Author	Data collection, data analysis, critical revision of the article
Colt D. Capan	Van Andel Institute, Center For Neurodegenerative Sciences, Grand Rapids, MI	Author	Data analysis, critical revision of the article
Amanda R. Burmeister, PhD	Van Andel Institute, Center For Neurodegenerative Sciences, Grand Rapids, MI	Author	Critical revisions of the manuscript, including statistical calculations.
Guangwei Du, MD, PhD	Penn State University, Milton S. Hershey Medical Center, Hershey, PA	Author	Study concept and design, development of MRI methodology, MRI data analysis, critical revision of the article
Martha L. Escobar Galvis, PhD	Van Andel Institute, Center For Neurodegenerative Sciences, Grand Rapids, MI	Author	Study concept and design, critical revision of the article
Patrik Brundin, MD, PhD	Van Andel Institute, Center For Neurodegenerative Sciences, Grand Rapids, MI	Author	Study concept and design, critical revision of the article
Xuemei Huang, MD, PhD	Penn State University, Milton S. Hershey Medical Center, Hershey, PA	Author	Study concept and design, clinical examination of patients, data analysis, critical revision of the article, obtaining funding
Lena Brundin, MD, PhD	Van Andel Institute, Center For Neurodegenerative Sciences, Grand Rapids, MI	Author	Study concept and design, data analysis, review of statistical analysis, drafting the initial manuscript, obtaining funding
