# Supplementary material for: Tryptophan Metabolites Are Associated With Symptoms and Nigral Pathology in Parkinson's Disease
Source: Mov Disord. 2020 Jul 25;35(11):2028–37. doi: 10.1002/mds.28202 (PMC7754343; doi:10.1002/mds.28202)
Supplement: Supplementary file 1 — SUPPLEMENTAL TABLE 1 Subject demographics. MoCA, Montreal Cognitive Assessment; UPSIT, University of Pennsylvania Smell Identification Test; R2*, relaxation rates in the substantia nigra pars compacta; N/A; not available. All values are reported as mean ± SD.1 aStudent t test. bChi‐square test. cANCOVA, with age and sex as covariates. [file MDS-35-2028-s001.docx]

**Supplemental Table 1: Subject Demographics.**

|  | **HC (n=90)** | **PD (n=97)** | **Significance** |
| --- | --- | --- | --- |
| Age (Years) | 66.6 ± 10.3 | 66.9 ± 8.3 | p = 0.79^a^ |
| Sex (M/F) | 43/47 | 49/48 | p = 0.70^b^ |
| BMI | 28.1 ± 4.8 | 27.5 ± 5.0 | p = 0.38^a^ |
| Education (Years) | 15.3 ± 2.6 | 14.8 ± 2.6 | p = 0.13^a^ |
| Disease Duration (Years) | N/A | 6.5 ± 6.5 | N/A |
| UPDRS I | 3.6 ± 3.8 | 9.7 + 7.2 | **p < 0.001^c^** |
| UPDRS II | 0.45 ± 0.97 | 10.3 ± 9.6 | **p < 0.001^c^** |
| UPDRS III | 4.3 ± 4.3 | 26.9 + 19.1 | **p < 0.001^c^** |
| MoCA | 25.4 ± 2.4 | 23.8 +3.8 | **p = 0.001^c^** |
| UPSIT | 32.1 ± 6.5 | 19.2 ± 7.5 | **p < 0.001^c^** |
| R2* | 25.7  (23.3-28.2) | 27.7  (24.7-30.0) | **p < 0.05 ^d^** |
