# Supplementary material for: Tryptophan Metabolites Are Associated With Symptoms and Nigral Pathology in Parkinson's Disease
Source: Mov Disord. 2020 Jul 25;35(11):2028–37. doi: 10.1002/mds.28202 (PMC7754343; doi:10.1002/mds.28202)
Supplement: Supplementary file 2 — SUPPLEMENTAL TABLE 2 Median levels of metabolites and inflammatory markers. All values are reported as Median (IQR). aMann‐Whitney U test. [file MDS-35-2028-s002.docx]

**Supplemental Table 2. Biomarkers in plasma and CSF**

| 1. **Plasma Analyte Levels (Median, IQR)** | | | |
| --- | --- | --- | --- |
| **Analyte** | **HC (N = 89)** | **PD (N = 97)** | ***p*-value** **^a^** |
| **3-HK [nM]** | 32.7 (27.1-42.8) | 44.0 (32.1-60.0) | **0.000005*** |
| **3-HAA [nM]** | 18.0 (12.4-23.4) | 14.0 (10.2-20.3) | **0.008*** |
| **NTA [nM]** | 360.3 (280.4-498.2) | 297.0 (234.3-449.3) | **0.05*** |
| PIC [nM] | 38.4 (28.5-47.7) | 32.4 (25.9-47.6) | 0.12 |
| KYNA [nM] | 42.7 (34.1-56.5) | 40.6 (30.7-51.5) | 0.22 |
| KYN [mM] | 2.2 (1.9-2.6) | 2.2 (1.8-2.5) | 0.37 |
| QUIN [nM] | 398.9 (313.6-525.1) | 366.6 (316.1-470.1) | 0.39 |
| TRP [mM] | 55.7 (48.4-63.5) | 55.0 (49.2-61.0) | 0.84 |
| **CRP (mg/mL**) | 3.2 (1.3-8.0) | 1.7 (0.6-3.5) | **0.001*** |
| SAA (mg/mL) | 5.3 (2.9-1.3) | 4.1 (2.3-8.5) | 0.083 |
| 1. **CSF Analyte Levels** | | | |
| **Analyte** | **HC (N = 30)** | **PD (N = 25)** | ***p*-value** **^a^** |
| **KYNA [nM]** | 3.3 (2.2-4.7) | 2.1 (1.6-3.2) | **0.029 *** |
| TRP [mM] | 2.9 (2.4-3.5) | 3.5 (2.7-3.9) | 0.073 |
| PIC [nM] | 16.1 (13.8-21.6) | 17.4 (15.2-23.2) | 0.28 |
| 3-HK [nM] | 4.45 (3.1-8.03) | 3.7 (2.6-6.4) | 0.28 |
| QUIN [nM] | 45.1 (35.2-55.2) | 38.8 (29.8-55.3) | 0.40 |
| NTA [nM] | 21.8 (12.3-46.1) | 28.0 (12.6-37.2) | 0.72 |
| KYN [nM] | 57.1 (49.6-77.9) | 60.4 (51.1-75.3) | 0.81 |
| SAA (mg/mL) | 0.3 (0.2-0.7) | 0.2 (0.2-0.4) | 0.07 |
| CRP (mg/mL) | 1.2 (0.4-3.2) | 0.6 (0.4-2.2) | 0.33 |
| 1. **Kynurenine Metabolite Ratios** | | | |
| **Ratio** | **HC (N = 30)** | **PD (N = 25)** | ***p*-value** **^a^** |
| CSF KYN/TRP | 0.022 (0.014-0.030) | 0.018 (0.015-0.022) | 0.19 |
| **CSF KYNA/KYN** | 0.055 (0.037-0.068) | 0.034 (0.028-0.047) | **0.002*** |
| **CSF QUIN/KYNA** | 14.4 (9.4-19.7) | 20.3 (14.1-24.1) | **0.046*** |
| CSF QUIN/3HK | 9.4 (4.6-15.6) | 9.3 (6.9-13.8) | 0.73 |
| CSF QUIN/PIC | 2.8 (1.9-3.8) | 2.1 (1.6-3.0) | 0.084 |
