# Supplementary material for: Tryptophan Metabolites Are Associated With Symptoms and Nigral Pathology in Parkinson's Disease
Source: Mov Disord. 2020 Jul 25;35(11):2028–37. doi: 10.1002/mds.28202 (PMC7754343; doi:10.1002/mds.28202)
Supplement: Supplementary file 3 — SUPPLEMENTAL TABLE 3 Correlations over the blood–brain barrier (BBB) Pearson correlation coefficients and P values after adjusting for sex and age. FDR‐adjusted P values are listed in separate columns. [file MDS-35-2028-s003.docx]

**Supplemental Table 3: Correlations over the BBB (adjusted for sex and age)**

| **Biomarker** | | **PD PATIENTS** | | **CONTROLS** | |
| --- | --- | --- | --- | --- | --- |
|  |  | **R,**  **p-value** | **FDR** | **R,**  **p-value** | **FDR** |
| **Z-score**  **Plasma CRP** | **CSF**  **QUIN** | **0.52**  **0.015*** | **0.043*** | 0.075  0.70 | 0.84 |
| **Z-score Plasma SAA** | **CSF**  **QUIN** | **0.50**  **0.02*** | **0.048*** | -0.25  0.21 | 0.40 |
| **Z-score Plasma CRP** | **CSF KYN/TRP** | **0.57**  **0.007*** | **0.027*** | 0.18  0.36 | 0.59 |
| **Z-score Plasma SAA** | **CSF KYN/TRP** | **0.52**  **0.016*** | **0.043*** | 0.032  0.87 | 0.93 |
| **Z-score Plasma CRP** | **CSF QUIN/KYNA** | 0.12  0.59 | 0.76 | -0.11  0.57 | 0.76 |
| **Z-score Plasma SAA** | **CSF QUIN/KYNA** | 0.15  0.50 | 0.75 | -0.31  0.10 | 0.22 |
| **Z-score Plasma CRP** | **CSF QUIN/PIC** | **0.56**  **0.008*** | **0.027*** | 0.06  0.75 | 0.86 |
| **Z-score Plasma SAA** | **CSF QUIN/PIC** | **0.77**  **0.00005*** | **0.0002*** | -0.18  0.37 | 0.59 |
| **Z-score Plasma CRP** | **CSF 3-HK** | -0.017  0.94 | 0.94 | -0.032  0.87 | 0.93 |
| **Z-score Plasma SAA** | **CSF 3-HK** | -0.12  0.60 | 0.76 | -0.23  0.23 | 0.43 |
| **Z-score Plasma CRP** | **CSF CRP** | **0.90**  **0.000005*** | **0.00005*** | **0.76**  **0.000002** | **0.00039*** |
| **Z-score Plasma SAA** | **CSF SAA** | **0.82**  **0.000005*** | **0.00005*** | **0.66**  **0.000006*** | **0.00005*** |

CSF = Cerebral spinal fluid; CRP = C-reactive Protein; QUIN = Quinolinic acid; SAA = Serum Amyloid Alpha; KYN = Kynurenine; TRP = Tryptophan; KYNA = Kynurenic acid; PIC = Picolinic acid; 3-HK = 3-Hydroxykynurenine
