# Supplementary material for: Tryptophan Metabolites Are Associated With Symptoms and Nigral Pathology in Parkinson's Disease
Source: Mov Disord. 2020 Jul 25;35(11):2028–37. doi: 10.1002/mds.28202 (PMC7754343; doi:10.1002/mds.28202)
Supplement: Supplementary file 4 — SUPPLEMENTAL TABLE 4 Correlation of biological analytes with disease duration in years. Pearson correlation coefficients and P values after adjusting for sex and age. FDR‐adjusted P values are listed in separate columns. [file MDS-35-2028-s004.docx]

**Supplemental Table 4: Correlation of disease duration vs biomarkers**

| **Correlation disease duration vs biomarker**  **Z-scores** | **R** | **p-value** | **FDR**  **p-value** |
| --- | --- | --- | --- |
| **Plasma 3-HK** | **0.33** | **0.001*** | **0.009*** |
| **CSF CRP** | **0.51** | **0.01*** | **0.056*** |
| **CSF SAA** | **0.71** | **0.000075*** | **0.001*** |
| **Plasma 3-HAA** | -0.095 | 0.35 | 0.68 |
| **Plasma QUIN** | 0.036 | 0.73 | 0.83 |
| **Plasma PIC** | -0.11 | 0.27 | 0.68 |
| **Plasma KYNA** | -0.070 | 0.49 | 0.69 |
| **Plasma KYN/TRP** | 0.071 | 0.49 | 0.69 |
| **Plasma QUIN/KYNA** | 0.19 | 0.07 | 0.30 |
| **Plasma CRP** | -0.007 | 0.95 | 0.95 |
| **Plasma SAA** | 0.059 | 0.57 | 0.69 |
| **CSF 3-HK** | -0.030 | 0.89 | 0.95 |
| **CSF QUIN** | 0.19 | 0.35 | 0.68 |
| **CSF PIC** | -0.17 | 0.40 | 0.68 |
| **CSF KYNA** | 0.19 | 0.37 | 0.68 |
| **CSF KYN/TRP** | 0.31 | 0.14 | 0.48 |
| **CSF QUIN/KYNA** | -0.12 | 0.57 | 0.69 |

3-HK = 3-Hydroxykynurenine; CSF = Cerebral spinal fluid; CRP = C-reactive Protein; SAA = Serum Amyloid Alpha; 3-HAA = 3-Hydroxyanthranilic acid; QUIN = Quinolinic acid; PIC = Picolinic acid; KYNA = Kynurenic acid; KYN = Kynurenine; TRP = Tryptophan
